# PSMA PET/CT in biochemical recurrence of prostate cancer with PSA levels ≤ 0.2 ng/mL: a German multicenter analysis of conventional PSMA tracers, including [^68^Ga]Ga-PSMA-11, [^68^Ga]Ga-PSMA I&T, and [^18^F]PSMA-1007

**DOI:** 10.1007/s00259-025-07292-1

**Published:** 2025-04-30

**Authors:** Caroline Burgard, Madita Frei, Arne Blickle, Philipp E. Hartrampf, Manuela A. Hoffmann, Mathias Schreckenberger, Hans-Peter Schmid, Lena Unterrainer, Julian Rogasch, Markus Galler, Samer Ezziddin, Florian Rosar

**Affiliations:** 1 Department of Nuclear Medicine, Saarland University– Medical Center, Kirrberger Str. 100, Geb. 50, D-66421 Homburg, Germany; 2 Department of Nuclear Medicine, University Hospital Würzburg, Würzburg, Germany; 3 Department of Nuclear Medicine, Johannes Gutenberg University– Medical Center, Mainz, Germany; 4 Department of Nuclear Medicine, Ludwig Maximilian University of Munich– University Hospital, Munich, Germany; 5 Bavarian Cancer Research Center (BZKF), Partner Site Munich, Munich, Germany; 6 Charité– Universitätsmedizin Berlin, corporate member of Freie Universität Berlin and Humboldt-Universität zu Berlin, Berlin, Germany

**Keywords:** PSMA, PET/CT, PSA, Detection rate, Biochemical recurrence, PSMA-11, PSMA I&T, PSMA-1007

## Abstract

**Background:**

Prostate-specific membrane antigen (PSMA)-directed positron emission tomography/computed tomography (PET/CT) has emerged as a highly accurate imaging modality for detecting tumor lesions in patients with biochemical recurrence (BCR) of prostate cancer (PC). While detection rates of lesions suspicious for PC relapse are known to increase with rising prostate-specific antigen (PSA) levels, data on the efficacy of PSMA PET/CT at very low PSA values (≤ 0.2 ng/mL) remain limited.

**Methods:**

In this multicenter study, we analyzed 321 patients with BCR and a PSA value ≤ 0.2 ng/mL across five German academic centers, using three different PSMA-targeted radiotracers: [^68^Ga]Ga-PSMA-11, [^68^Ga]Ga-PSMA I&T, and [^18^F]PSMA-1007 and analyzed the detection rates and potential predictive parameters.

**Results:**

The overall pooled detection rate was 29.6%. No statistically significant differences in detection rates were observed between the three radiotracers ([^68^Ga]Ga-PSMA-11 29.4% vs. [^68^Ga]Ga-PSMA I&T, 22.5% vs. [^18^F]PSMA-1007 32.4%, *p* ≥ 0.314). Detection rates were significantly higher in patients with a PSA level > 0.15 ng/mL (*p* = 0.029, φ = 0.122), in those with an initial Gleason score > 7 (*p* = 0.018, φ = 0.141) and in those receiving androgen deprivation therapy (*p* = 0.031, φ = 0.120).

**Conclusion:**

All three radiotracers demonstrated comparable diagnostic performance, with no significant superiority observed between the ^68^Ga- and ^18^F-labeled tracers in the patient sample investigated (overall pooled detection rate: 29.6%). This positivity rate can serve as an expectation horizon for both the attending physician and the patient in the case of low PSA values. Further studies with larger cohorts, preferably conducted in a prospective setting, are needed to confirm and expand upon our findings.

**Supplementary Information:**

The online version contains supplementary material available at 10.1007/s00259-025-07292-1.

## Introduction

The prostate-specific membrane antigen (PSMA), has emerged as a key target in modern diagnostics and therapy of prostate cancer (PC) [1–5], which is one of the most prevalent malignancies in men worldwide [6]. PSMA is overexpressed on the surface of these tumor cells, and thus represents a promising target in both, imaging and treatment [7–9]. PSMA-directed positron emission tomography/computed tomography (PET/CT) with different tracers such as [^68^Ga]Ga-PSMA-11, [^68^Ga]Ga-PSMA I&T or [^18^F]PSMA-1007 has been established in diagnostics, especially in primary staging and in the localization of biochemical recurrence (BCR) enabling a highly accurate and early localization of the tumor lesions [10–13]. It is well known that the detection rate of lesions suspicious for prostate cancer using [^68^Ga]Ga-PSMA-11 PET/CT increases with rising blood levels of prostate-specific antigen (PSA) [14, 15]. However, data on the sensitivity for very low levels of < 0.2 ng/mL is scarce, with only a few studies including > 100 patients [16, 17]. Similarly, the sensitivity of other radiotracers, like [^68^Ga]Ga-PSMA I&T or [^18^F]PSMA-1007 for low PSA values was rarely addressed [13, 18]. In addition to the sparse literature, inconsistent results, limited generalizability, and lack of direct comparisons result in difficulties assessing or predicting detection rates in very low PSA settings. In order to contribute to this clinically important issue, our group participated in a two-center study in 2023 with retrospective real world analysis from a sample of 115 men demonstrating that [^68^Ga]Ga-PSMA-11 PET/CT has the potential to be a valuable diagnostic tool in the context of BCR with very low PSA [19]. The prompt detection of tumor lesions can enable the initiation of targeted therapy, potentially improving patient outcomes and possibly delaying the need for side effect-prone systemic ADT. The current study presents an update on this highly relevant issue, now including *n* = 321 patients examined by PSMA PET/CT at 5 different institutions, aiming to further evaluate performance of three different conventional radiotracers, which are applied in clinical routine in Germany ([^68^Ga]Ga-PSMA-11, [^68^Ga]Ga-PSMA I&T, [^18^F]PSMA-1007) in the context of BCR with very low PSA levels ≤ 0.2 ng/mL.

## Methods

### Study design and patient characteristics

The data from *n* = 321 patients with a history of radical prostatectomy and increasing but very low blood PSA concentration ≤ 0.2 ng/mL were retrospectively extracted from registers of the Saarland University Medical Center, Homburg (*n* = 70), the University Hospital Würzburg (*n* = 121), the Johannes Gutenberg-University Medical Center, Mainz (*n* = 56; in cooperation with the Practice for Radiology and Nuclear Medicine Köln-Triangle, Cologne), the Charité Universitätsmedizin Berlin (*n* = 48) and the University Hospital– Ludwig Maximilian University of Munich (*n* = 26). Patients were referred for PSMA PET/CT by their treating urologist and PSA concentration was assessed by the respective local laboratories. According to the inclusion criteria patients were enrolled in the study if they showed an increase in PSA levels, with the concentration remaining ≤ 0.2 ng/mL. Furthermore, PET/CT images following treatment by radical prostatectomy had to be available generated with either the radiotracer [^68^Ga]Ga-PSMA-11, [^68^Ga]Ga-PSMA I&T or [^18^F]PSMA-1007. Patient characteristics are summarized in Table 1. In the course of disease, a minority of patients had received salvage radiation (*n* = 67) or androgen deprivation therapy (*n* = 67), in addition to radical prostatectomy. According to Gleason scores obtained from previous tissue samples (data available for *n* = 283 patients), the majority of patients had an intermediate (Gleason score = 7) or high risk (Gleason score ≥ 8). At the time of PET/CT imaging, PSA concentration was < 0.1 ng/mL in 28.0% of patients, 0.1–0.15 ng/mL in 25.6% of patients and > 0.15 (maximum 0.2 ng/mL) in 46.4% of patients. PSA doubling time (dt) was available for 149 patients, with a median of 5.45 months and a range of 0.75 to 29.2 months.

**Table 1:** Patient characteristics

Patient Characteristic	Value
Age (*n* = 321)	
Median	68.0
Range	48.4–85.7
Mean ±SD	67.8 ± 7.53
PSA (*n* = 321)	
Median	0.15
Range	0.005-0.20
Mean ± SD	0.137 ± 0.057
Category, ng/mL, % (n)	
<0,1	28.0% (90)
0,1 − 0,15	25.6% (82)
>0,15 − 0,2	46.4% (149)
PSA Doubling Time, months (*n* = 149)	
Median	5.45
Range	0.75–29.2
Category, months, % (n)	
<3	23.5% (35)
3–6	30.9% (46)
>6–12	28.9% (43)
>12	16.8% (25)
Initial Gleason Score (*n* = 283)	
Range	6–10
6	6.7% (19)
7	55.5% (157)
8	14.1% (40)
9	21.6% (61)
10	2.1% (6)
Treatments (*n* = 321)	
Radical prostatectomy	100% (321)
Salvage radiation therapy	20.9% (67)
ADT	20.9% (67)

Primary endpoint of the analysis was the detection rate of any lesions suspicious for prostate cancer, defined as the ratio of positive scans to total scans. Secondary endpoints were the detection rate separated by the applied radiotracer, blood PSA concentration, PSA doubling time (dt), initial Gleason score and previous therapies.

Patients agreed to anonymized publication of any resulting data in accordance with the Declaration of Helsinki. The analysis was approved by the authorized and responsible ethics committees.

### PET/CT imaging procedures

All patients underwent whole body scanning using positron emission tomography / computed tomography (PET/CT) ~ 60–90 min after intravenous injection of the respective tracer. Patients received either [^68^Ga]Ga-PSMA-11 (*n* = 170) or [^68^Ga]Ga-PSMA I&T (*n* = 40) or [^18^F]PSMA-1007 (*n* = 111) PET/CT. Administered activities and performed procedures adhered to the recommendations of EANM guidelines [20]. Details of the PET/CT imaging procedures can be found in the supplementary material (Table S1). All PET/CT scanners used for imaging were accredited by the EANM Research GmbH (EARL) initiative.

### Statistical analysis

Patient data, imaging data characteristics and scan findings are displayed as descriptive statistics as applicable. All statistical tests were performed using the Prism software, version 9 (GraphPad software, San Diego, CA, USA). Statistical significance was defined by *p*-value < 0.05. The Fisher’s exact test was applied to compare overall detection rates of suspicious lesions among subgroups, addressing the metric scales factors of blood PSA value and PSA doubling time (dt), while the Chi-square test was used addressing the ordinal scaled Gleason score, pretreatment, and applied tracer. The phi coefficient φ was employed as a means of quantifying the strength of association between the detection rates and binarized categories for the aforementioned variables.

## Results

**Fig. 1:**
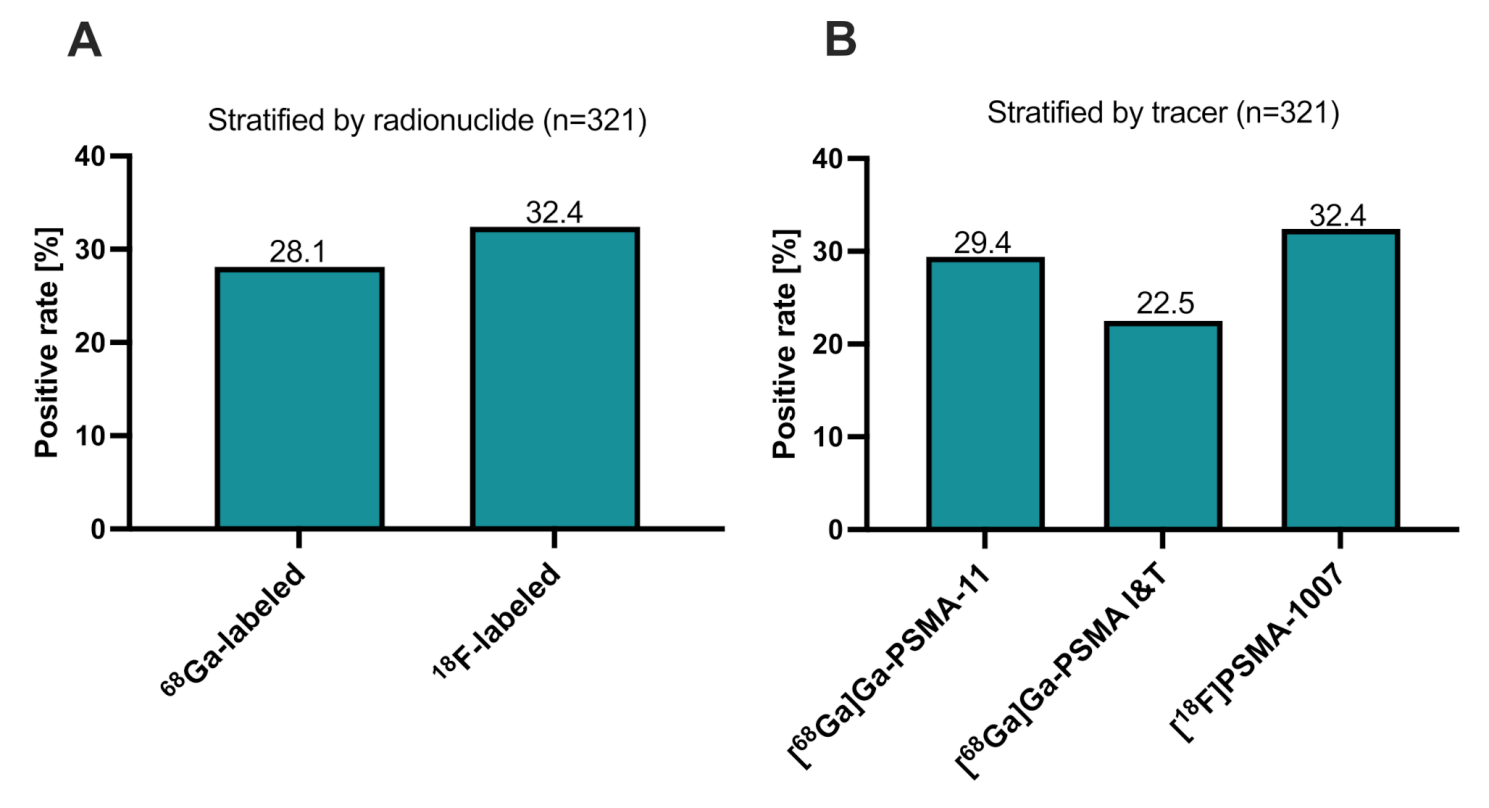
**A**) Comparison of the positivity rates regarding the applied radionuclide and **B**) the three different radiotracers

**Fig. 2:**
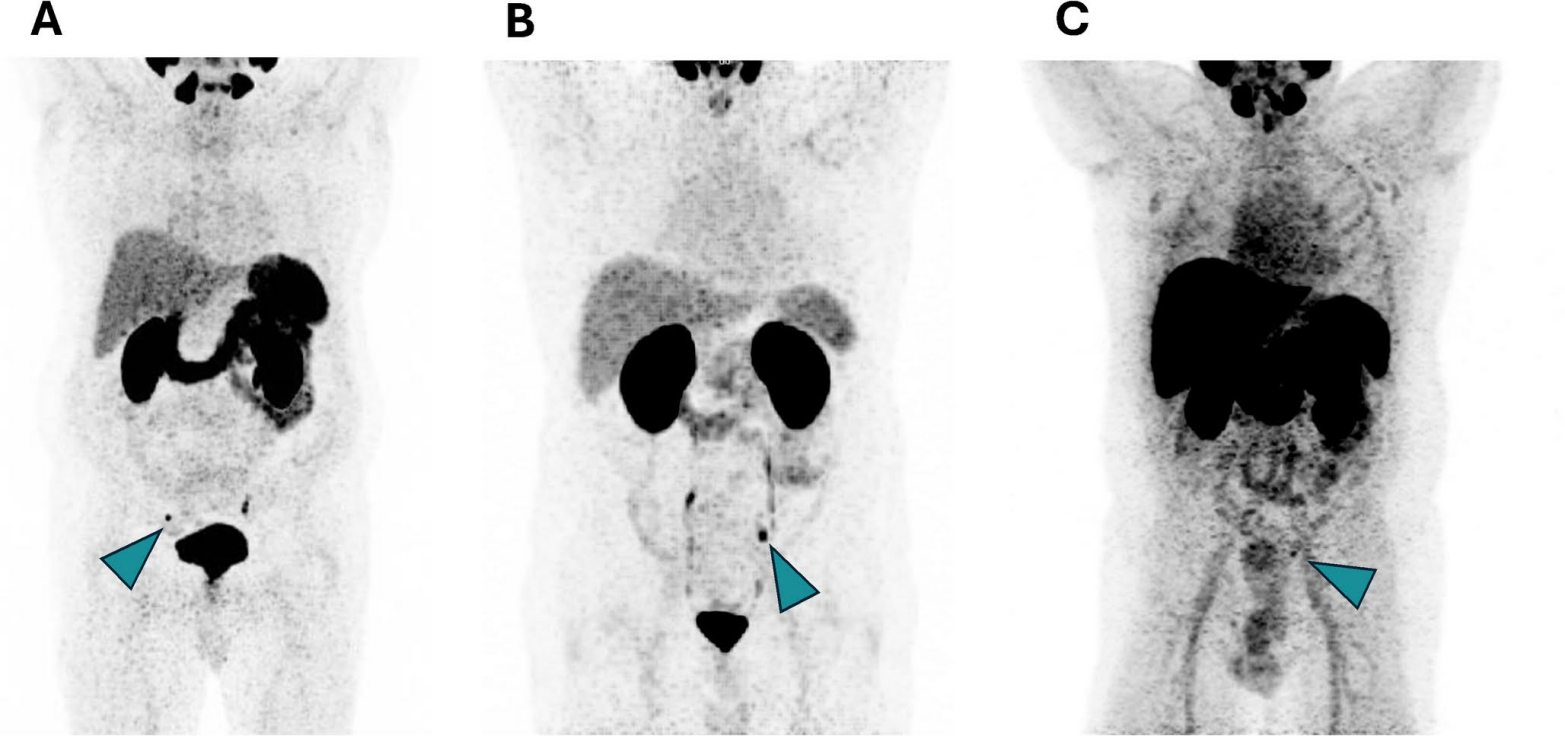
Maximum intensity projections of positive PSMA PET/CT scans with successful localization of BCR (lymph node metastasis) using the three different radiotracers **A**) [^68^Ga]Ga-PSMA-11, **B**) [^68^Ga]Ga-PSMA I&T and **C**) [^18^F]PSMA-1007. Blue arrows indicate the localization of BCR

For the total cohort of 321 patients with BCR and a PSA value ≤ 0.2 ng/mL, the overall detection rate of PSMA-targeted PET/CT was 29.6% (95/321 patients). Figure 1 presents the positivity rate stratified by applied PSMA tracer. For the ^68^Ga-labeled PSMA tracers the detection rate was comparable to the ^18^F-labeled PSMA-tracer (28.1% vs. 32.4%, *p* = 0.442). Specifically, for [^68^Ga]Ga-PSMA-11, the detection rate was 29.4%, for [^68^Ga]Ga-PSMA I&T 22.5% and for [^18^F]PSMA-1007 32.4%. Pair-wise differences in detection rates between the tracers were not statistically significant ([^68^Ga]Ga-PSMA-11 vs. [^68^Ga]Ga-PSMA I&T *p* = 0.439; [^68^Ga]Ga-PSMA-11 vs. [^18^F]PSMA-1007 *p* = 0.599; [^68^Ga]Ga-PSMA I&T vs. [^18^F]PSMA-1007 *p* = 0.314). Figure 2 depicts a representative example of a positive PSMA-PET/CT scan for each tracer successfully localizing BCR. The measured SUV_max_ values, categorized by the applied tracer and by localization of the suspicious lesions, are shown in the supplementary material (Table S2).

**Fig. 3:**
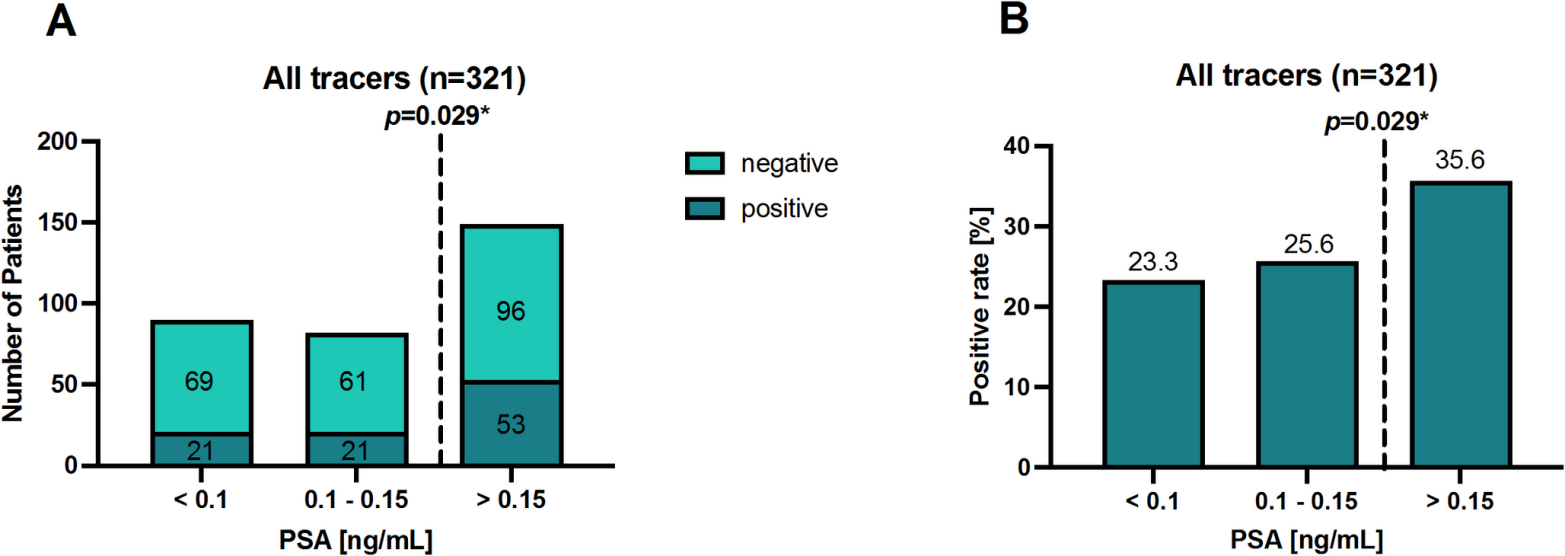
**A**) The numbers of patients with positive and negative PSMA PET/CT and **B**) the corresponding detection rates categorized according to the patients’ PSA level

The proportions of positive/negative scans and the corresponding detection rates separated by different levels of blood PSA are depicted in Fig. 3. For PSA levels < 0.1 ng/mL a detection rate of 23.3% (21/90 patients) was recorded. In case of slightly higher PSA levels between 0.1 and 0.15 ng/mL, the detection rate was 25.6% (21/82 patients), while for PSA levels > 0.15 ng/mL the detection rate significantly increased (*p* = 0.029) to 35.6% (53/149 patients).

**Fig. 4:**
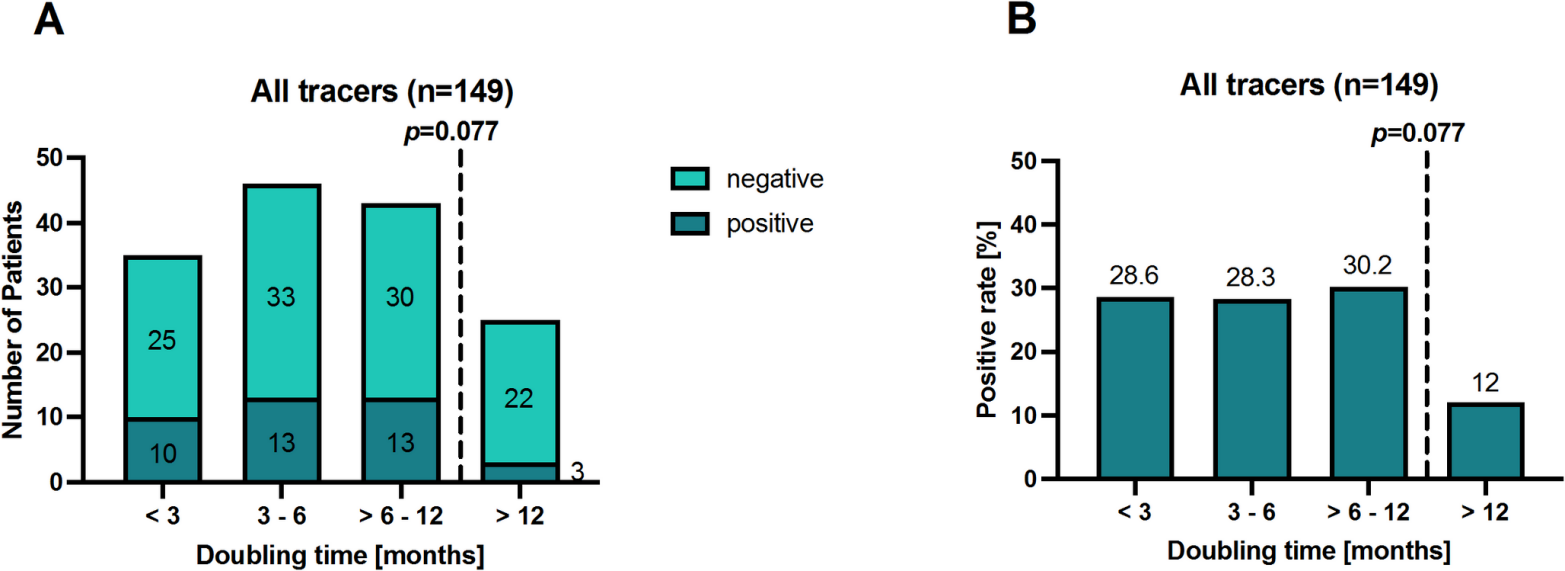
**A**) The numbers of patients with positive and negative PSMA PET/CT scans, and **B**) the corresponding positivity rates categorized according to the patients’ PSA doubling time

For 149 of 321 patients (46.4%), PSA doubling time (dt) was available. The number of positive and negative PET/CT scans, along with the detection rate, were analyzed with regard to PSA dt (Fig. 4). A detection rate of 28.6% (10/35 patients) was found for a PSA dt < 3 months, similar to 28.3% (13/46 patients) for a PSA dt between 3 and 6 months and 30.2% (13/43 patients) for a PSA dt > 6–12 months. No statistically significant difference was found comparing these combined groups with the group of patients showing a PSA dt of more than 12 months (*p* = 0.077), although the detection rate (3/25 patients, equaling 12.0%) was notably lower for those patients with PSA dt > 12 months.

**Fig. 5:**
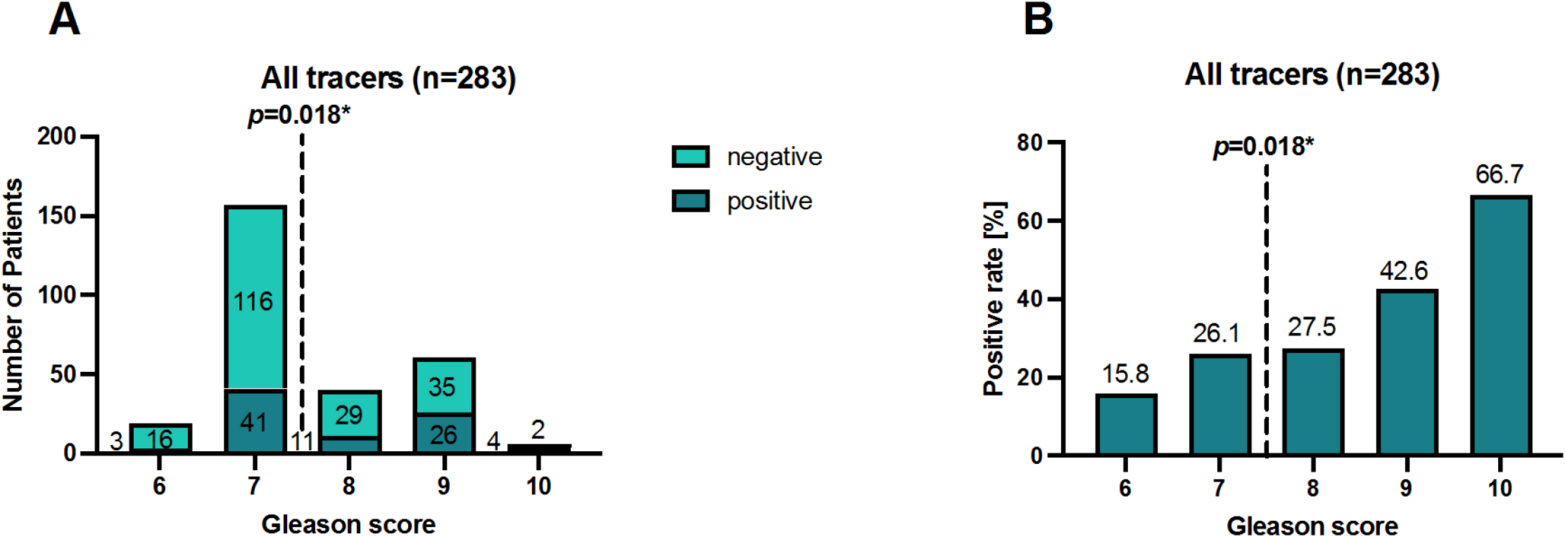
**A**) The numbers of patients with positive and negative PSMA PET/CT scans, and **B**) the corresponding positivity rates categorized according to the patients’ initial Gleason score

For 283 of the 321 patients (88.2%) data regarding the initial Gleason score was available and analyzed. Figure 5 illustrates detection rates according to initial Gleason score categories. The detection rate was 15.8% (3/19 patients) and 26.1% (41/157 patients) for initial Gleason score 6 and 7, respectively, while for Gleason scores 8 or higher, the detection rate was significantly increased (*p* = 0.018). Specifically, detection rates of 27.5% (11/40 patients), 42.6% (26/61 patients) and 66.7% (4/6 patients) were found for initial Gleason score 8, Gleason score 9 and Gleason score 10 respectively.

Table 2 presents the calculated positivity rates, along with the corresponding statistical significance (*p*-value) and effect size (φ coefficient) for each of the aforementioned parameters as well as for additionally analyzed patient characteristics such as previous treatment with ADT or radiotherapy. Summarizing, blood PSA (*p* = 0.029), initial Gleason score (*p* = 0.018) and preceding treatment with ADT (*p* = 0.031) were identified as statistically significant parameters associated with the detection rate of lesions suspicious for PC. All three parameters, PSA value (φ = 0.122), Gleason score (φ = 0.141) and ADT as a preceding therapy (φ = 0.120) showed a slightly positive association with the lesion detection rate. No statistically significant impact on the detection of suspicious lesions was found for PSA dt (*p* = 0.077; φ=−0.145) and prior radiation-treatment (*p* = 0.340; φ = 0.053).

**Table 2:** Association between binarized clinical parameters with the positivity rate of PSMA PET/CT imaging

Category	Threshold	Positivity Rate [%]	*p*-Value	φ Coefficient
PSA	≤ 0.15 > 0.15	24.42 35.57	**0.029**	0.122
PSA dt	≤ 12 > 12	29.03 12.00	0.077	–0.145
Gleason Score	≤ 7 > 7	25.00 38.32	**0.018**	0.141
Salvage Radiation	Yes No	34.33 28.35	0.340	0.053
ADT	Yes No	40.30 26.77	**0.031**	0.120

## Discussion

This retrospective analysis, including 321 patients, represents to the best of our knowledge the largest cohort to date assessing the efficiency of PSMA-targeted PET/CT in detecting biochemical recurrence (BCR) of prostate cancer with very low PSA levels ≤ 0.2 ng/mL. Conducted in a multicenter setting across five academic research institutions, the study evaluated and compared the detection rates of three commonly used radiotracers in Germany: [^68^Ga]Ga-PSMA-11, [^68^Ga]Ga-PSMA I&T, and [^18^F]PSMA-1007. The key finding was the overall detection rate for all three PSMA-targeting radiotracers pooled together was 29.6%. No statistically significant differences in positivity rates were observed between [^68^Ga]Ga-PSMA-11 (29.4%), [^68^Ga]Ga-PSMA I&T (22.5%), and [^18^F]PSMA-1007 (32.4%) (*p*-values ≥ 0.314). A similar detection rate of 25.2% for [^68^Ga]Ga-PSMA-11 was reported in a previous study of our group in a smaller cohort of patients with PSA levels ≤ 0.2 ng/mL [19]. These reported positivity rates can serve as an expectation horizon for both the attending physician and the patient in the case of very low PSA values. However, other studies investigating detection rates in comparable settings have reported varied results, as summarized in a meta-analysis by De Visschere et al. [21]. For example, Meredith et al. observed a notably lower positivity rate of 11.3% in patients with PSA levels between 0.01 and 0.2 ng/mL [22], while Hope et al. reported a much higher detection rate of 58.3% [23]. The largest current study on PSMA-PET/CT imaging, conducted by Afshar-Oromieh et al. on 2533 men with BCR, found a detection rate of 43% in a subgroup of 226 patients with PSA levels ≤ 0.2 ng/mL [16].

Similarly, Hoffmann et al. reported a detection rate of 41% for [^68^Ga]Ga-PSMA-11 in a subgroup of 37 patients with BCR and PSA levels < 0.2 ng/mL [24]. Notably, Wang et al. recently analyzed the detection rate of [^68^Ga]Ga-PSMA-11 in BCR patients with PSA < 0.2 ng/mL, reporting a detection rate of 73.75% using total body PET/CT and 43.75% with conventional PET/CT [25]. In conclusion, the literature presents a mixed picture regarding the detection rates of PSMA-targeted imaging in patients with BCR and very low PSA levels. For the interpretation and comparison of the presented studies, differences in imaging protocols, patient selection criteria, scanner, and patient preparation must be considered, as they potentially influence the outcome of the respective analysis.

When comparing the three radiotracers — [^68^Ga]Ga-PSMA-11, [^68^Ga]Ga-PSMA I&T, and [^18^F]PSMA-1007 — we found no evidence of superiority for any one of them in the setting of very low PSA values. This contrasts with a study by Solomonidou et al. on PET/CT-guided salvage therapy, which suggested a higher detection rate using [^18^F]PSMA-1007 compared to [^68^Ga]Ga-PSMA-11. In their study of 273 patients, Solomonidou et al. reported a pooled detection rate of 43.2%, with [^18^F]PSMA-1007 showing a significantly higher detection rate than [^68^Ga]Ga-PSMA-11 (odds ratio 2.2) [26]. However, our findings align with other studies indicating no significant difference between the three radiotracers. For instance, Berliner et al. compared [^68^Ga]Ga-PSMA I&T with previously published data on [^68^Ga]Ga-PSMA-11 and found no difference in detection rates between the two tracers [27]. Similarly, Gühne et al. conducted a study including a comparison between [^68^Ga]Ga-PSMA-11 and [^68^Ga]Ga-PSMA I&T and reported no superiority of one radiotracer, neither in patients with PSA ≤ 0.1 ng/mL nor PSA ≤ 0.5 ng/mL [28]. Hoffmann et al. compared [^68^Ga]Ga-PSMA-11 and [^18^F]PSMA-1007 in a cohort of patients undergoing PET/CT for primary staging and also found no significant difference in detection rates, concluding that both tracers are equally effective for staging primary prostate cancer [29]. In addition, a systematic review and network meta-analysis by Alberts et al. showed no superiority of one tracer [30].

We identified three parameters with slightly positive association with lesion detection. The analysis revealed that the positivity rates were significantly higher for patients with a PSA level of > 0.15 ng/mL at the time of imaging (*p* = 0.029, φ = 0.122) and in those with a Gleason score > 7 (*p* = 0.018, φ = 0.141), which is consistent with findings from previous research [19]. Additionally, prior treatment with ADT was associated with a higher positivity rate compared to patients who had not received ADT before (*p* = 0.031, φ = 0.120). This could be related to the presence of a more advanced disease stage. Although the calculated φ values indicate a statistically small positive effect size, suggesting a slight association with lesion detection, the clinical impact of this association may still be relevant. Potentially, these parameters could contribute to the improvement of patient selection in the future. For instance, PSA values greater than 0.15 ng/mL and a Gleason score above 7 are considered suitable for this purpose, with potential detection rates of more than 40%.

In conclusion, the results of this study demonstrate that the commonly used PSMA-targeting radiotracers, specifically [^68^Ga]Ga-PSMA-11, [^68^Ga]Ga-PSMA I&T, and [^18^F]PSMA-1007, show comparable detection rates. The analysis found no evidence to suggest the superiority of either ^68^Ga- or ^18^F-labeled tracers, and there was no significant difference observed in the performance of the three radiotracers. The observed positivity rate can serve as a benchmark for both the attending physician and the patient when dealing with low PSA values. However, in order to achieve even higher detection rates, long-lived PSMA-targeted tracers are currently being discussed. Promising results have already been reported in pilot studies, particularly with regard to the detection of positive findings in cases that were previously negative on conventional PSMA PET/CT [31–34]. These early findings point to the potential for improved diagnostic accuracy and highlight the value of these advanced radiotracers in clinical practice. Further studies with larger cohorts, preferably in a prospective setting, are needed to confirm and expand upon the findings in the future.

It is important to acknowledge the limitations of this study, including its retrospective design and the varying sizes of the sub-cohorts, which may have influenced the results. Additionally, not all of the 321 patients provided the complete set of laboratory values and clinical data, leading to discrepancies in cohort sizes for the subsequent analyses (no imputation performed). A further limitation is the lack of multiparametric analysis. Additionally, it should be noted that inter-observer agreement was not assessed. However, while the imaging protocols and scanner technology varied across the five participating institutions, it is worth noting that all centers were EARL-accredited, ensuring a standardized level of quality in the imaging procedures.

## Conclusion

In the setting of biochemical recurrence (BCR) with PSA levels ≤ 0.2 ng/mL, this multicenter study of 321 patients demonstrated a pooled detection rate with PSMA PET/CT of 29.6%. Differences in detection rates between the ^68^Ga- and ^18^F-labeled tracers ([^68^Ga]Ga-PSMA-11, [^68^Ga]Ga-PSMA I&T, and [^18^F]PSMA-1007) were not significant. This positivity rate can serve as an expectation horizon for both the attending physician and the patient in the case of low PSA values. Further studies with larger cohorts, preferably conducted in a prospective setting, are needed to confirm and expand upon our findings.

## Electronic supplementary material

Below is the link to the electronic supplementary material.

Supplementary Material 1: Table S1: Technical information for PET/CT imaging procedures of the five different institutions.

Supplementary Material 2: Table S2: SUVmax values categorized by localization of suspicious lesion and applied tracer.

## Data Availability

The datasets used and analyzed during the present study are available from the corresponding author upon reasonable request.

## Abbreviations

ADT: Androgen Deprivation Therapy
BCR: Biochemical Recurrence
dt: Doubling Time
EANM: European Association of Nuclear Medicine
EARL: European Association of Nuclear Medicine Accreditation of Radiopharmaceuticals and PET/CT Systems
PC: Prostate Cancer
PET/CT: Positron Emission Tomography/Computed Tomography
PSA: Prostate-Specific Antigen
PSMA: Prostate-Specific Membrane Antigen
SUV: Standardized Uptake Value

## Declarations

### Transparency statement

Parts of the cohort analyzed here were originally described in a previous publication by our group.
