# Supplementary material for: PSMA PET/CT in biochemical recurrence of prostate cancer with PSA levels ≤ 0.2 ng/mL: a German multicenter analysis of conventional PSMA tracers, including [68Ga]Ga-PSMA-11, [68Ga]Ga-PSMA I&T, and [18F]PSMA-1007
Source: Eur J Nucl Med Mol Imaging. 2025 Apr 30;52(12):4368–76. doi: 10.1007/s00259-025-07292-1 (PMC12491085; doi:10.1007/s00259-025-07292-1)
Supplement: Supplementary file 1 — Supplementary Material 1: Table S1: Technical information for PET/CT imaging procedures of the five different institutions. [file 259_2025_7292_MOESM1_ESM.docx]

**Table S1:** Technical information for PET/CT imaging procedures of the five different institutions.

| **PET Parameters** | **Saarland University Medical Center Homburg** | **University Hospital Ludwig Maximilian University of Munich** | **Johannes Gutenberg University Medical Center**  **(in collaboration with the Radiology and Nuclear Medicine Practice at the Köln Triangle, Cologne)** | **Charité Universitätsmedizin Berlin** | **University Medical Center Würzburg** |
| --- | --- | --- | --- | --- | --- |
| Tracer | [^68^Ga]Ga-PSMA-11 | [^18^F]PSMA-1007 | [^68^Ga]Ga-PSMA-11  [^18^F]PSMA-1007 | [^68^Ga]Ga-PSMA-11 | [^68^Ga]Ga-PSMA I&T  [^18^F]PSMA-1007 |
| Time: injection to imaging [min] | ~60 min | ~60 min | [^68^Ga]Ga-PSMA-11:  ~ 60 min  [^18^F]PSMA-1007:  ~ 90 min | ~60 min | [^68^Ga]Ga-PSMA I&T:  ~ 60 min  [^18^F]PSMA-1007:  ~90 min |
| PET/CT scanner Model | Siemens Biograph  mCT 40 | GE Discovery  690 | Philips Gemini  TF 16 | Philips Gemini  TF 16 | Siemens Biograph  mCT 128 flow |
| Pixel matrix size | 200x200 | 256x256 | 168x168 | 144x144 | 200x200 |
| Acquisition time/bed position [min] | 2 min | 2 min | 2 min. | 3 min | 2 min |
| Extended field of view [cm] | 21.4 cm | 15.4 cm | 21.8 cm | 18.0 cm | 78.0 cm |
| Slice thickness [mm] | 3.0 mm | 3.27 mm | 3.0 mm | 4.0 mm | 3.0 mm |
| **Reconstruction Parameters** |  |  |  |  |  |
| No. of iterations | 3 | 3 | 2 | 3 | 3 |
| No. of subsets | 21 | 36 | 14 | 33 | 21 |
| Reconstructed slice thickness [mm] | 5.0 mm | 5.0 mm | 4.0 mm | 4 mm | 5.0 mm |
| Low dose CT / Diagnostic CT | Low dose CT | Diagnostic CT | Low-dose CT | Low dose CT | Low-dose CT |
