# Supplementary material for: PSMA PET/CT in biochemical recurrence of prostate cancer with PSA levels ≤ 0.2 ng/mL: a German multicenter analysis of conventional PSMA tracers, including [68Ga]Ga-PSMA-11, [68Ga]Ga-PSMA I&T, and [18F]PSMA-1007
Source: Eur J Nucl Med Mol Imaging. 2025 Apr 30;52(12):4368–76. doi: 10.1007/s00259-025-07292-1 (PMC12491085; doi:10.1007/s00259-025-07292-1)
Supplement: Supplementary file 2 — Supplementary Material 2: Table S2: SUVmax values categorized by localization of suspicious lesion and applied tracer. [file 259_2025_7292_MOESM2_ESM.docx]

**Table S2:** *SUV_max_ values categorized by localization of suspicious lesion and applied tracer.*

|  | **[^68^Ga]Ga-PSMA-11** | **[^68^Ga]Ga-PSMA-I&T** | **[^18^F]PSMA-1007** |
| --- | --- | --- | --- |
| Positivity Rate | 50/170 patients (29.4%) | 9/40 patients (22.5%) | 36/111 patients (32.4%) |
| Median PSA (baseline) | 0.14 ng/mL | 0.17 ng/mL | 0.16 ng/mL |
| Range PSA (baseline) | 0.01 − 0.2 ng/mL | 0.01 − 0.2 ng/mL | 0.005 − 0.2 ng/mL |
| All Lesions |  |  |  |
| Median | 5.7 | 8.1 | 6.5 |
| Range | 1.3-33.1 | 4.6-18.0 | 3.1-43.4 |
| Mean ±SD | 8.3 ± 7.3 | 8.6 ± 3.6 | 8.1 ± 7.0 |
| Local Recurrence |  |  |  |
| Median | 4.3 | 6.5 | 7.0 |
| Range | 1.3-19.4 | 5.3-10.8 | 2.0-43.4 |
| Mean ±SD | 5.8 ± 4.8 | 7.6 ± 2.4 | 9.4 ± 9.5 |
| Lymph node metastases |  |  |  |
| Median | 7.1 | 8.9 | 6.0 |
| Range | 2.1-33.1 | 4.6-18.0 | 2.5-28.5 |
| Mean ± SD | 9.1 ± 6.7 | 10.1 ± 5.3 | 8.3 ± 6.7 |
| Bone metastases |  |  |  |
| Median | 4.2 | 8.2 | 5.5 |
| Range | 2.3-31.0 | 5.0-14.4 | 3.5-16.9 |
| Mean ± SD | 9.4 ± 9.8 | 8.5 ± 2.8 | 6.7 ± 3.2 |
